# Supramolecular Systems for Gene and Drug Delivery

**DOI:** 10.3390/pharmaceutics14030471

**Published:** 2022-02-22

**Authors:** José A. Lebrón, Pilar López-Cornejo, Francisco J. Ostos

**Affiliations:** 1 Department of Physical Chemistry, Faculty of Chemistry, University of Seville, C/Profesor García González 1, 41012 Seville, Spain; jlebron@us.es; 2 Clinical Unit of Infectious Diseases, Microbiology and Preventive Medicine, Institute of Biomedicine of Seville (IBiS), Virgen del Rocío University Hospital, CSIC, University of Seville, 41013 Seville, Spain; 3 Department of Medical Biochemistry, Molecular Biology, and Immunology, School of Medicine, University of Seville, 41009 Seville, Spain

Several biomaterial-based supramolecular systems (cyclodextrins [1], calixarenes [2,3], polymers [4], carbon nanotubes [5], nanoparticles [6,7], liposomes [3,8], nanogels [9], and nanocomplexes [10], among others) have been widely used for biomedical applications, such as gene and drug delivery. Numerous researchers have developed novel supramolecular systems for enhancing their biocompatibility and pharmacological activity, thus increasing their therapeutic properties. These nanosystems are considered to be promising platforms in gene therapy and drug delivery due to their higher transfection (or encapsulation) efficiency and low cytotoxicity.

This Special Issue, “Supramolecular Systems for Gene and Drug Delivery”, brings together the latest research articles, published in *Pharmaceutics*. Noticeably, 10 original research articles were published by authors from 12 different countries on what is a hot topic in this research field.

I. Asela et al. [1] prepared nanosponges based on β-cyclodextrin (βCDNS), which were loaded with the drugs phenylethylamine (PhEA) and 2-amino-4-(4-chlorophenyl)-thiazole (AT). Subsequently, the supramolecular βCDNS drug complexes were functionalized with gold nanoparticles (AuNPs), forming the βCDNS-PhEA-AuNP and βCDNS-AT-AuNP systems. The drug-loading capacity was higher for the βCDNS and βCDNS-drug-AuNP systems than with native βCD.

B. Gómez-González et al. [2] studied the formation of inclusion complexes between alkyl sulfonate guests and a cationic pillar [5] arene receptor in water using NMR and ITC measurements. The results demonstrated the formation of host–guest complexes stabilized by electrostatic interactions and hydrophobic effects.

J. A. Lebrón et al. [3] studied the formation of calixarene-based liposomes. Four amphiphilic calixarenes were used. The lipid bilayer was formed with one calixarene and with the phospholipid 1,2-dioleoyl-sn-glycero-3-phosphoethanolamine (DOPE). The liposomes containing the least cytotoxic calixarene (TEAC_12_)_4_ were used as nanocarriers of both nucleic acids and the antineoplastic drug doxorubicin (DOX). The results showed that (TEAC_12_)_4_/DOPE/p-EGFP-C1 lipoplexes, of a given composition, can transfect the genetic material, although the transfection efficiency substantially increases in the presence of an additional amount of DOPE as coadjuvant. On the other hand, the (TEAC_12_)_4_/DOPE liposomes showed a high doxorubicin encapsulation efficiency and a slow controlled release, which could diminish the side effects of the drug.

V. Karava et al. [4] prepared microparticles (MPs) based on newly synthesized poly(l-lactic acid)-co-poly(butylene adipate) (PLA/PBAd) block copolymers for the preparation of aripiprazole (ARI)-loaded long-acting injectable (LAI) formulations. In terms of in vitro dissolution profile, results suggested that the newly synthesized PLA/PBAd block copolymers can successfully control the release rate and extent of the API’s release from the prepared MPs, indicating that, probably, under in vivo conditions, their use may lead to new formulations that will be able to maintain a continuous therapeutic level for an extended period of time, with reduced lag time compared to the currently marketed ARI LAI product.

L. Tang et al. [5] successfully prepared a multi-walled carbon nanotube (MWNT)-based drug delivery system with the synergistic effect of PTT photothermal therapy and chemotherapy for efficient tumor removal. The integration of photothermal agents ICG-NH2 to MWNT was achieved by linking hyaluronic acid (HA). To realize the synergistic therapeutic effect of chemotherapy and phototherapy, DOX was attached on the wall of MWNT via a π–π interaction to obtain the final MWNT-HA-ICG/DOX nanocomplexes. Both in vitro and in vivo experiments verified the great therapeutic efficacy of MWNT-HA-ICG/DOX nanocomplexes.

L. S. Mbatha et al. [6] formulated folic acid (FA)-modified, poly-amidoamine-generation-5 (PAMAM G5D)-grafted gold nanoparticles (AuNPs) and evaluated their cytotoxicity and transfection efficiency using the luciferase reporter gene (FLuc-mRNA) in vitro. These nanosystems showed low cytotoxicity and good transfection efficiency.

S. Yin et al. [7] prepared NPs based on the insertion of two types of functional peptides, half-life extension peptide PAS and tumor-targeting peptide RGDK (Arg-Gly-Asp-Lys), into human heavy-chain ferritin (HFn) at the C-terminal through flexible linkers with two distinct enzyme-cleavable sites. RGDK peptide enhanced the internalization efficiency of HFn and showed a significant increase in growth inhibition. Pharmacokinetic study in vivo demonstrated that PAS peptides extended ferritin half-life. RGDK peptides greatly enhanced drug accumulation in the tumor site, rather than in other organs, in a biodistribution analysis. Drug-loaded, PAS-RGDK-functionalized HFns curbed tumor growth with significantly greater efficacies in comparison with drug-loaded HFn. 

C. E. Torres et al. [8] prepared magnetoliposomes (MLP), which are liposomes that contain magnetite nanoparticles (MNP) inside. This study presents a low-cost microfluidic approach for the synthesis and purification of MLPs to improve their biocompatibility, with functional testing via hemolysis, platelet aggregation, cytocompatibility, internalization, and endosomal escape assays to determine their potential application in gastrointestinal delivery. In addition, the authors achieved encapsulation efficiencies between 20% and 90% by varying the total flow rates (TFRs), flow rate ratios (FRRs), and MNP concentrations.

F. Bintang Ilhami et al. [9] developed a new concept in cooperative adenine–uracil (A-U) hydrogen bonding interactions between anticancer drugs and nanocarrier complexes, which was successfully demonstrated by invoking the co-assembly of water-soluble, uracil end-capped polyethylene glycol polymer (BU-PEG) upon association with the hydrophobic drug adenine-modified rhodamine (A-R6G). This concept holds promise as a smart and versatile drug delivery system, which leads to the formation of self-assembled A-R6G/BU-PEG nanogels in aqueous solution, for the achievement of targeted, more efficient cancer chemotherapy. 

A. Jagusiak et al. [10] described the Congo red–doxorubicin (CR-DOX) complexes, analyzed their interaction with some proteins, and explained the mechanism of this interaction. This kind of interaction between CR-DOX and the described proteins may in future become an important therapeutic system, with the possibility of targeted drug transport and delivery. Supramolecular ribbon-like CR complexed with doxorubicin is a promising system in the treatment of cancers and may open new avenues for novel treatment strategies.

We would like to thank all the authors and reviewers of this Special Issue. We also acknowledge the Assistant Editor, Ms. Daisy Tu, for her tremendous efforts in ensuring its implementation. In addition, authors are encouraged to submit original research articles and reviews in the next Special Issue, “Supramolecular Systems for Gene and Drug Delivery (Volume II)”, led by us.
